# The Relationship between Personality, Motivation and Academic Performance at Medical Students from Romania

**DOI:** 10.3390/ijerph19158993

**Published:** 2022-07-24

**Authors:** Lorena Mihaela Muntean, Aurel Nireștean, Andreea Sima-Comaniciu, Marius Mărușteri, Cătălin Andrei Zăgan, Emese Lukacs

**Affiliations:** 1 Department of Psychiatry, George Emil Palade University of Medicine, Pharmacy, Science, and Technology of Targu Mures, 540136 Targu Mures, Romania; lorena-mihaela.muntean@umfst.ro (L.M.M.); emese.lukacs@umfst.ro (E.L.); 2 Department of Medical Informatics and Biostatistics, George Emil Palade University of Medicine, Pharmacy, Science, and Technology of Targu Mures, 540136 Targu Mures, Romania; marius.marusteri@umfst.ro; 3 County Emergency Clinic Hospital of Targu Mures, Neurosurgery Clinic, 540136 Targu Mures, Romania; andrei.zag96@gmail.com

**Keywords:** personality, motivation, medical students, academic achievements, academic performance

## Abstract

The academic and health system requirements are constantly growing due to the continuous development of this sector. Therefore, it is important to investigate the structural factors that improve performance in the medical system. The aim of our pilot study is to analyze if there are associations or correlations between personality and motivation and the results obtained for the National Residency Exam of Romanian medical graduates. We conducted a prospective pilot study on 179 medical students from George Emil Palade University of Medicine, Pharmacy, Science, and Technology of Targu Mures, Romania between February 2021 and December 2021, who were evaluated by the DECAS, IM, and SPM scale. Our results showed that all the dimensions of personality according to the Big Five Model, which include openness (OR = 0.392, *p* = 0.01), extraversion (OR = 0.512, *p* = 0.03), conscientiousness (OR = 3.671, *p* = 0.004), agreeableness (OR = 2.791, *p* = 0.07), and emotional stability (OR = 4.863, *p* = 0.0003), are statistically associated with the result obtained. Motivation also plays an important role in academic achievements, through motivational persistence and motivational involvement which correlates with the conscientiousness dimension and the result obtained. This study confirms that both personality structure and motivation are associated or correlated with the academic results of medical students and represent a starting point for future research.

## 1. Introduction

In the last years, several research studies on academic performance have been conducted, focusing on the factors that have an influence on the development of the medical student. These factors include intellectual capacity, personality, motivation, but also environmental factors related to lifestyle which depends on economic and social support [1]. Current medical practice enjoys an increase in the quality of services provided and professionalism, but in order for these to happen, efforts are needed on the part of both students and teachers [2].

Each student is unique in the way they view, analyze, process, organize, and learn information presented by the faculty [3]. Academic success is significantly affected by individual structure, with motivation playing a major role [4]. Most of the time, the student’s persistence and effort has a major impact on academic success, which is why it is important to investigate the predictors of academic success [5].

In order to become a doctor in Romania, medical graduates must pass the National Residency Exam after which, depending on the score obtained, the graduate can choose the medical specialty that they want to practice. Annually in Romania the number of candidates registered at the exam increases, from 4533 in 2016 to 6396 in 2020. In 2021, the total number of candidates was 5756 and the number of places was 3942 [6].

Motivation is a dynamic, complex structure that manifests [7] in both personal and professional life [8], determining our behavior to meet our needs or the desire to achieve a certain goal [9]. According to the theory of self-determination developed by Deci and Ryan, people are motivated to develop through innate abilities that are influenced by personality traits but also by personal well-being [10]. In the same context, motivation is an independent variable in medical education that has an impact in academic results, being determined by autonomy, competence, and the need for interpersonal interactions [11].

Academic motivation is a concept that shows the correlation between student motivation and academic success and several factors have a great impact on it: student expectations, teachers’ attitudes, conditions for teaching activities, and personal structure [12]. Pintrich et al. identified three factors that can influence academic motivation: the belief that the subject has the necessary skills to complete the action, the reasons why the subject is involved in the activity, and how the subject feels performing this action [13]. Motivation issues have a significant impact on academic performance [12]. Medical students are motivated by the desire to become doctors, which may be due to social recognition, interest in knowledge and understanding, but also in the need to increase self-esteem through this achievement [13]. In order to succeed in medicine, the level of motivation must be high [14].

Motivation has two sub-variants: intrinsic and extrinsic motivation. Intrinsic motivation refers to the inner desire to perform a certain action, while extrinsic motivation refers to the fact that the action is performed due to the result or the reward that can be obtained or the appreciation of others [15,16]. Increased intrinsic motivation is associated with better orientation and learning ability. Low intrinsic and extrinsic motivation scores are associated with low academic achievement [17]. In a study by Buddeberg-Fischer et al., a group of 1004 medical students showed that men had higher values for extrinsic motivation, being interested in income, prestige and career advancement, as opposed to women who have higher values for intrinsic motivation [18]. Another recent study shows that there is a strong correlation between intrinsic motivation and professional identity, which shows that academic motivation adjusts behavior for success and plays an important role in developing professional identity through deeper learning, superior performance, and increased creativity [19].

It is known that personality influences the performance of students and doctors [20]. From a dimensional point of view, according to the Big Five model (Five Factor model), five dimensions are described: openness, extraversion, conscientiousness, agreeableness, and emotional stability [21]. Lievens et al. demonstrated that medical students have higher scores on extraversion dimension and agreeableness than other students [1]. The dimension of conscientiousness has a positive impact on the academic success aspect demonstrated by several authors in their research. Ferguson et al. demonstrated that increased conscientiousness leads to superior performance in structured, orderly, and methodical activities; otherwise, if the activity requires flexibility, the performance will decrease [22]. The extraversion dimension also has an impact on academic achievements [23]. Bhagat et al. demonstrated that low emotional stability has a negative effect on academic achievement [24]. Both the emotional stability dimension and conscientiousness dimension contribute to the level of stress felt during university. If the stress level is high, it will lead to decreased ability to concentrate, including decreased academic performance [25].

A theory related to academic performance is the one developed by Walberg according to which academic productivity depends on the personal structure (ability, development, motivation), teaching (both the amount of information taught and its clarity) and the environment where the student lives and carries out his activities (home, classroom, peer group outside the classroom and mass media) [26,27]. 

In light of the studies mentioned above, and the novelty in the field of our country, the purpose of our pilot study is to investigate if a relationship exists between personality, motivation, and the result of the National Residency Exam. It is the first study carried out comparing the result obtained at a National Exam in Romania, held over a period of 3 h for which the candidates have previously prepared a certain period of time, from a common bibliography of 850 pages. The study is also a novelty, due to the attempt to see if the conscientiousness dimension is related to the motivation of medical graduates. Given the above, we try to find: (1) if associations exist between the dimensions of personality and the score obtained at the exam of residency; (2) if openness and conscientiousness dimensions together can form a model which is associated to the result obtained at the National Residency Exam because they have the greatest impact on academic performance; (3) if correlations exist between intrinsic motivation, extrinsic motivation, and the result obtained; and (4) if there is a link between the conscientiousness dimension and the factors of motivational persistence.

## 2. Materials and Methods

### 2.1. Study Design

We conducted a prospective pilot study on medical students from the last year at the George Emil Palade University of Medicine, Pharmacy, Science, and Technology of Targu Mures. The study took place between February 2021 and December 2021. The research was approved by Ethics Committee of the George Emil Palade University of Medicine, Pharmacy, Science, and Technology of Targu Mures by decision no. 1250/28.01.2021. All the subjects signed the informed consent form before enrolling in the study.

### 2.2. Participants and Procedure

Between February 2021 and June 2021, subjects completed the following scales: DECAS Personality Inventory, Motivational Involvement Scale (IM), and Motivational Persistence Scale (SPM). In December 2021, the subjects were contacted after taking the National Residency Exam to communicate the score obtained and the chosen specialty. In addition to the applied scales, the following parameters were included in the study: age, sex, score obtained, and chosen specialty. 

The total number of students graduating in 2021 from the Faculty of Medicine within the George Emil Palade University of Medicine, Pharmacy, Science, and Technology of Targu Mures is 241 [28]. 

Therefore, in our pilot study, we included 179 students from a total of 214 students who completed the scales voluntary. A number of 35 participants were excluded due to the fact that they did not meet the eligibility criteria for participation. A total of 19 students were excluded from the study because the internal validation scales of the DECAS personality questionnaire indicated distortions of the answers, 6 students could not be contacted in order to communicate the score and chosen specialty, and 10 students did not take the residency exam. 

Inclusion criteria: (1) Students who are in the 6th year at the Faculty of Medicine of the George Emil Palade University of Medicine, Pharmacy, Science, and Technology of Targu Mures, and (2) Students who passed the National Residency Exam. 

Exclusion criteria: (1) Students who obtained distorted answers by evaluating the internal validation scales of the DECAS Personality Inventory, (2) Students who could not be contacted in order to communicate the obtained score and chosen specialty, and (3) Students who have not been in the final year.

The participants signed the informed consent form before responding to the study questionnaires. The questionnaires were disseminated online through social media to the group of medical students from the 6th year, graduates of the 2021 Promotion from the Faculty of Medicine of the George Emil Palade University of Medicine, Pharmacy, Science, and Technology of Targu Mures.

### 2.3. Measures

#### 2.3.1. Personality

The DECAS Personality Inventory evaluates the personality according to the Big Five model. The tool developed by Sava et al. is validated on the Romanian population, being composed of 97 statements that evaluate the following dimensions: openness, extraversion, conscientiousness, agreeableness, and emotional stability [21]. The openness dimension describes issues related to intellectual concerns, culture, curiosity, aspirations, and high standards. The extraversion dimension is found in all models and refers to aspects related to social interactions, optimism, energy, but also self-confidence. The conscientiousness dimension refers to the desire for assertion, ambition, organization and structure, perseverance, but also the capacity for self-control. The agreeableness dimension refers to altruism, modesty, the ability to cooperate, to work in a team, to be oriented towards the needs of others, but also to have confidence in others. The emotional stability dimension refers to self-control, control of emotions in conflictual situations, but also vulnerability. The tool also contains three internal validation scales to determine distorted responses. The social desirability validation scale—the subject tries to look better; random answers validation scale—the subject gives random answers; and approval validation scale—the subject tends to answer most of the questions with “true” or “false”. The internal consistency of the DECAS personality inventory was measured on a sample of 1552 subjects. The Alpha Cronbach coefficient for the openness dimension is 0.71, 0.75 for the extraversion dimension, 0.70 for the conscientiousness dimension, 0.71 for the agreeableness dimension, and 0.75 for the emotional stability dimension [21]. The values obtained are interpreted as follows: 20–35—very low values, 35–45—low values, 45–55 medium values, 55–65—high values, and 65–80—very high values [21]. The value set for performing statistical tests and interpretation of the results is 45 because this value represents the threshold limit between a low and a medium value at each dimension.

#### 2.3.2. Academic Motivation

The Motivational Involvement Scale (IM) is an autochthonous scale developed by Constantin et al., according to the model of Leonard, Beauvais, and Scholl. The scale is validated on the Romanian population being a good tool for identifying the preferences but also the personal motivations that underlie the choice of school or professional objectives and also the degree of involvement. The scale measures motivational involvement through the following four dimensions: (1) hedonistic involvement—pleasure—the subject is motivated by pleasant tasks, without external pressures, (2) social involvement—recognition—motivation comes from recognition of the entourage, (3) internalist involvement—challenge—the subject is motivated by challenges, difficult tasks which offers self-validation, and (4) instrumental involvement—reward—motivation comes from concrete, material rewards or those that help in hierarchical promotion. To determine the scores of intrinsic motivation and extrinsic motivation, the four dimensions will be calculated together. The intrinsic motivation consists of hedonistic and internalist involvement—the subject is involved in the activity due to curiosity, challenge, but also pleasure. Extrinsic motivation formed by the sum of the dimensions of social and instrumental involvement is the motivation that derives from the quality of the rewards, the advantages obtained, but also the appreciation received from others. The scale consists of 44 items rated on a dichotomous scale with answers “yes” or “no”. The internal consistency was determined by applying the questionnaire to a number of 756 subjects of Romanian nationality with the Alpha Cronbach between 0.65 and 0.83 for dimensions [28,29,30].

The Motivational Persistence Scale (SPM) was developed by Constantin et al. and aims to assess the motivational persistence which is seen as the individual predisposition to persist in the activity initiated but also to find the motivation to achieve the set goals. Evaluation is done by the following six factors: ambition—setting ambitious goals, determination—pursuing long-term goals, planning—preparing daily activities, implementation—accomplishing daily tasks, recurrence—recalling unattainable goals, and self-discipline—monitoring and self-constraint. The instrument consists of 30 items scored on a five-point Likert scale from 1 = to a very low level, to 5 = to a very high level. The Alpha Cronbach coefficient on the initial version of the questionnaire was measured on a group of 762 subjects from the Romanian population ranging between 0.75 and 0.86. The questionnaire is validated for the Romanian population [29,31,32,33].

#### 2.3.3. Academic Performance

Academic performance is represented by the score obtained at the National Residency Exam. Given the fact that based on this result students can choose the desired specialty, we calculated the average of the last 4 years to determine the score that allows the choice of any specialty at the national level [34,35,36,37]. This score represents the value 752.

### 2.4. Statistical Analysis

Statistical analysis was performed using Graph Pad Prism 9, Microsoft Office Excel, and MedCalc software. The statistical significance threshold was set at 0.05, with a confidence interval CI of 95%. The statistical analysis included elements of descriptive statistics (mean, median, standard deviation) and elements of inferential statistics. To test the normality of the data distribution, we used the Kolmogorov–Smirnov test. To test the significance of the association between the score and personality dimensions, we used the Chi^2^ test and Chi^2^ test with Yates’ Correction. All the statistical tests mentioned above were performed using the Graph Pad Prism 9 software. We used MedCalc software to perform logistic regression to evaluate the relationship between variables (score, openness, and conscientiousness dimension). To measure the strength and direction of the correlation between motivation, score, and conscientiousness dimension, we used Spearman test for nonparametric data. Microsoft Office Excel was used to create the database.

## 3. Results

Out of an initial number of 214 subjects who completed the scales, 179 students were included in the study because they met the eligibility criteria, of whom 133 (74.30) were women and 46 (25.70) were men. The total number of students from 6th year was 241 of which 214 answered; the non-response rate was 11.2%. Of the 179 students included, 6 could not be contacted to find out the chosen specialty; the non-response rate of those was 3.35%. The demographic characteristics of the participants can be found in Table 1.

The results mentioned in Table 2 tell us that the chance that a student with low openness will get a higher grade is 3.92 times higher and the effect size is medium (d = −0.516). Moreover, the chance that a student with low extraversion will get a better grade is 5.12 times higher, with a small effect size (d = −0.369). The chance of a student with a normal or high value of conscientiousness dimension is 3.671 times higher to obtain a higher grade with a fairly large effect size (d = 0.717). Moreover, students with increased agreeableness are 2.791 times more likely to get a better grade on the National Residency Exam, with a medium effect size (d = 0.566), and students with emotional stability within normal or high limits have a chance to get a better grade of 4.86 times higher, with a large effect size (d = 0.872). The associations and effect size mentioned above are detailed in Table 2. 

Given the importance of the openness and conscientiousness dimensions in the learning process, the two dimensions have been closely studied. We performed a logistic regression to investigate if the dimensions of conscientiousness and openness are associated together with the score obtained. The dependent variable is represented by the score, while the independent variables are openness and conscientiousness dimensions. We found a negative statistically significant association between openness dimension and score which means that the chance of subjects with low openness to get a better score is 3.36 times higher than others. We found a positive statistically significant association between the conscientiousness dimension and score which means that the chance of subjects with high conscientiousness to obtain a better score is 4.2 times higher. The model is statistically significant. The values discussed above are found in Table 3. 

In terms of motivation, we found a statistically significant positive correlation between persistence and conscientiousness dimension (r = 0.518, *p* < 0.0001). Among the factors of the SPM scale (ambition (r = 0.257, *p* = 0.0005), determination (r = 0.391, *p* < 0.0001), planning (r = 0.281, *p* = 0.0001), implementation (r = 0.332, *p* < 0.0001), self-discipline (r = 0.497, *p* < 0.0001)) and the conscientiousness dimension, we found statistically significant positive correlations, except for the recurrence factor (r = −0.045, *p* = 0.54) where no correlation was found. Moreover, between extrinsic motivation and conscientiousness (r = −0.211, *p* = 0.0046), we found a statistically significant negative correlation and between intrinsic motivation and conscientiousness (r = 0.16, *p* = 0.0249), a statistically significant positive correlation. Regarding the score and the factors ambition (r = 0.173, *p* = 0.02), implementation (r = 0.255, *p* = 0.0005), recurrence (r = −0.269, *p* = 0.0003), and self-discipline (r = 0.227, *p* = 0.002), we found statistically significant positive correlations. Regarding the motivational involvement, a statistically significant positive correlation was found between the intrinsic motivation and the score (r = 0.19, *p* = 0.0079). The study also showed a negative association between extrinsic motivation and the conscientiousness dimension (r = −0.211, *p* = 0.0046) and a positive correlation between intrinsic motivation and the conscientiousness dimension (r = 0.16, *p* = 0.0249). All the data mentioned are found in Table 4.

## 4. Discussion

Personality plays an important role in academic performance but also in academic motivation according to the latest studies in the field [38,39], so our study aimed to assess personality according to the Big Five Model in relation to the score obtained at the National Residency Exam by the graduates of the Faculty of Medicine from George Emil Palade University of Medicine, Pharmacy, Science, and Technology of Targu Mures.

Our results showed a positive association between the conscientiousness, agreeableness, and emotional stability dimensions and the result obtained, which are consistent with studies in the field [3,22]. We found a negative association between the openness dimension, extraversion dimension, and the academic performance. Regarding the extraversion dimension, there are studies which argue that there is no link between the extraversion dimension and academic performance [40,41] but there are also studies that show a positive correlation with academic results [42]. Students who have an increased value at extraversion dimension can more easily make friends to help them in the learning process, but they may also have more resources to help them in this process [43]. Our study showed that there is a negative association between the extraversion dimension and academic performance. Students whose extraversion dimension has low values are more self-oriented, quieter, are more attentive to what they have to accomplish professionally, prefer to learn on their own, and thus are more efficient, which are aspects that lead to better results [40,44,45]. Moreover, an important role is the environment in which the learning process takes place; in a highly stimulated environment with a lot of participants, extroverts will have better results while in a closed environment without external stimuli, their results may be affected [46]. Both agreeableness and emotional stability are positively associated with academic performance, the results being consistent with the research found in the literature [47]. Students with increased agreeableness are cooperative, supportive people able to work efficiently with others, which will lead to increased academic performance [48,49,50]. Moreover, students with high emotional stability dimension are calm, relaxed, and can focus in stressful situations, which will lead to better academic results than those who are anxious and have doubts about their knowledge [51].

Conscientiousness dimension is the most important predictor of the academic performance of medical students [52,53] along with the openness dimension, therefore they have important effects on academic performance [54], which is why we studied in detail the relationship between the two dimensions and the score obtained at the National Residency Exam. Hence, our findings revealed that the score obtained at the National Residency Exam by candidates was negatively associated to the openness dimension and positively associated to conscientiousness. Abdullatif et al. also demonstrated that conscientiousness is positively correlated with grade point average (GPA) [23] and the openness dimension in most studies was positively correlated with academic performance, which is the opposite of what we found [55,56]. Regarding the openness dimension, Gutierrez Tapia et al. demonstrated the same: the openness dimension correlates negatively with the academic performance [57]. Moreover, low openness and increased conscientiousness are associated with strategic learning [58].

Openness can be seen as a two-component construct consisting of sensory-aesthetic openness and intellectual openness [59]. For this reason, openness can have both positive and negative effects on academic performance [60]. Different facets such as openness to ideas and values are more relevant in the educational process than openness to feelings or actions that may have a negative effect on performance [61]. Openness to aesthetics, fantasy, and feelings will negatively affect academic performance [21,62]. Therefore, a negative association can often explain why a low level of openness corresponding to the facets oriented to aesthetics, feelings, and actions can have a positive effect on academic performance [61]. Increased levels of openness can lead to decreased ability to accumulate knowledge due to lack of discipline, determination, and people with high values who quickly become enthusiastic about various projects but do not find the resources to put them into practice [21,62]. Low levels, on the other hand, can lead to the non-recognition of opportunities in order to capitalize on them, but on the other hand the high practicality allows the appeal to the solutions already verified and efficient [21,62]. Regarding medical students, a lower openness allows easier memorization of information, which is often represented by clear definitions, guidelines, or procedures [60]. 

Conscientiousness is the most important dimension of the Big Five model by which academic performance is influenced [63,64], being the first dimension that was discovered to be closely related to academic motivation [65]. It is believed that increased conscientiousness can even compensate for lower intelligence, so students with lower intellectual ability and increased conscientiousness may have as good results as those with high intellectual capacity [66,67]. Positive correlations have been identified between the conscientiousness dimension and almost all motivational factors that ensure persistence in activity, demonstrating that conscientiousness is closely related to persistence in motivation [68]. This has also been demonstrated by Preckel et al., who considered that for academic failure, there is a linear relationship between motivation and conscientiousness [69]. Moreover, motivational persistence is correlated with the score. Fong et al. demonstrated that the more motivated the student is to persist in the activity, the score will increase [70]. There are other studies that confirm the effect that motivation has on education by planning activities in order to achieve academic success [71]. Students who enjoy academic success also have a higher level of conscientiousness and are more motivated [72].

This study shows a positive relationship between academic performance and intrinsic motivation. Students with high motivation are able to get involved more easily in activities [73,74]. They can put more effort for the accumulation of knowledge, being motivated by pleasure and the challenge they find inside them [75,76]. On the other hand, no relationship could be found between score and extrinsic motivation, according to which the subject is motivated by reward and social recognition [77]. Kuśnierz and Arbabisarjou confirmed the results [78,79]. Both intrinsic and extrinsic motivation correlate with conscientiousness, results that are similar to those of Clark et al. [80]. Students who are disciplined and organized are most likely more motivated to start different activities than the rest [4]. Conscientiousness dimension correlates negatively with extrinsic motivation, and Ariani obtained the same results [81]. The student is not motivated by reward and social recognition but by his/her way of being which determines him/her and maintain him/her in action [22].

## 5. Limitations

A possible limitation of the study could be the fact that in addition to the individual aspects represented by personality, motivation, and intellectual capacity, there are a number of external factors that can affect academic performance. These factors are represented by the environment in which the student carries out his daily activity referring to the particularities of the economic status but also to the family and social relationships and the habits developed by the student regarding the acquisition of information [82]. Therefore, we can show that personality and motivation are associated and corelated with academic performance in one direction, but we must always consider the factors mentioned above that may affect the results and are difficult to assess and interpret in the context of the study. Future studies may evaluate the environmental factors and the relationship between them and academic performance. Another limitation is represented by the fact that being a pilot study, the sample is small, only from a university, and also the fact that the participants were represented by 88.8% of the total number of students in year 6 through voluntary participation and not a random one which can lead to bias sampling. In the future, a study can be conducted in all medical universities from Romania, targeting medical graduates. Considering that the grade obtained was under conditions of stress being an exam carried out for three hours, in the future, comparative research could be done in which the students will be evaluated in several periods of time regarding the academic performance to compare the results. Due to the fact that it is a pilot study, the hypotheses generated require research in order to be validated in the future.

## 6. Conclusions

This study offers preliminary evidence regarding the relationship between personality structure, motivation, and academic results, more precisely the result obtained from the National Residency Exam by medical students from Romania. Moreover, a combination between low openness dimension and high conscientiousness dimension can predict a better grade at exam. Both motivational involvement and motivational persistence are correlated with the conscientiousness dimension and the score obtained at the National Residency Exam. In this context, these findings represent a starting point for future research and the knowledge of the structural features of the students’ personality allows the development of educational programs from which students can benefit from counseling to self-knowledge in order to take advantage of those dimensions and motivations that improve academic performance and are a source of permanent satisfaction and a major support of self-esteem.

## Figures and Tables

**Table 1:** Demographic characteristics.

Sample Characteristics	n = 179
Gender, n (%)	
Female	133 (74.30)
Male	46 (25.70)
Age range, M (SD)	23–35, 24.68 (1.33)
Choice of specialty, n (%)	
Medical	121 (67.60)
Surgical	28 (15.65)
Preclinical	24 (13.40)
None	6 (3.35)

Legend: M = mean; SD = standard deviation.

**Table 2:** Associations between personality dimensions and score.

DECAS	Score			
	OR	95% CI	*p*	Effect Size ***
Openness	0.392	0.190 to 0.810	0.01 *	−0.516
Extraversion	0.512	0.275 to 0.951	0.03 *	−0.369
Conscientiousness	3.671	1.523 to 8.847	0.004 **	0.717
Agreeableness	2.791	1.002 to 7.777	0.07 **	0.566
Emotional Stability	4.863	2.035 to 11.623	0.0003 **	0.872

Legend: OR = Odds Ratio; CI = Confidence Interval; * = Chi ^2^ test, *p* < 0.05 (two-tailed); ** = Chi ^2^ test with Yates correction, *p* < 0.05 (two-tailed), *** Cohen’s d.

**Table 3:** Logistic regression performed between openness and conscientiousness dimensions and score.

Independent Variables	B	Std. Error	Wald	*p*	OR	95% CI
Openness	−1.090	0.391	7.753	0.002	0.336	0.155 to 0.724
Conscientiousness	1.437	0.465	9.544	0.005	4.208	1.691 to 10.472

Legend: Independent variables: openness, conscientiousness; Dependent variable: score; B = coefficient; OR = Odds Ratio; CI = confidence interval.

**Table 4:** Correlations between SPM, IM, and Conscientious and Score.

	DECAS—Conscientiousness			Score		
	R	CI	*p* *	R	CI	*p* *
Ambition	0.257	0.110 to 0.393	0.0005	0.173	0.023 to 0.316	0.02
Determination	0.391	0.255 to 0.512	<0.0001	0.115	−0.036 to 0.261	0.124
Planning	0.293	0.148 to 0.425	<0.0001	0.09	−0.052 to 0.246	0.184
Implementation	0.332	0.190 to 0.460	<0.0001	0.255	0.109 to 0.391	0.0005
Recurrence	−0.045	−0.195 to 0.106	0.54	−0.269	−0.404 to −0.123	0.0003
Self-discipline	0.497	0.374 to 0.602	<0.0001	0.227	0.078 to 0.365	0.002
IM						
Extrinsic motivation	−0.211	−0.350 to −0.061	0.0046	−0.10	−0.248 to 0.050	0.178
Intrinsic motivation	0.16	0.017 to 0.310	0.0249	0.19	0.04 to 0.338	0.0079

Legend: SPM = The Motivational Persistence Scale; IM = The Motivational Involvement Scale; CI = Confidence Interval; * = Spearman test *p* < 0.05 (two-tailed).

## Data Availability

Not applicable.

## Abbreviations

DECAS	DECAS Personality Inventory
IM	The Motivational Involvement Scale
SPM	The Motivational Persistence Scale
